# Bivariate logistic regression model diagnostics applied to analysis of outlier cancer patients with comorbid diabetes and hypertension in Malawi

**DOI:** 10.1038/s41598-023-35475-z

**Published:** 2023-05-23

**Authors:** Tsirizani M. Kaombe, Jonathan Chiwanda Banda, Gracious A. Hamuza, Adamson S. Muula

**Affiliations:** 1 Department of Mathematical Sciences, School of Natural and Applied Sciences, University of Malawi, Zomba, Malawi; 2 Department of Public Health, Kamuzu University of Health Sciences, Blantyre, Malawi; 3 National Statistics Office, Zomba, Malawi

**Keywords:** Health care, Medical research, Risk factors

## Abstract

The joint occurrence of diabetes and hypertension conditions in a patient is common. The two diseases share a number of risk factors, and are hence usually modelled concurrently using bivariate logistic regression. However, the postestimation assessment for the model, such as analysis of outlier observations, is seldom carried out. In this article, we apply outlier detection methods for multivariate data models to study characteristics of cancer patients with joint outlying diabetes and hypertension outcomes observed from among 398 randomly selected cancer patients at Queen Elizabeth and Kamuzu Central Hospitals in Malawi. We used R software version 4.2.2 to perform the analyses and STATA version 12 for data cleaning. The results showed that one patient was an outlier to the bivariate diabetes and hypertension logit model. The patient had both diabetes and hypertension and was based in rural area of the study population, where it was observed that comorbidity of the two diseases was uncommon. We recommend thorough analysis of outlier patients to comorbid diabetes and hypertension before rolling out interventions for managing the two diseases in cancer patients to avoid misaligned interventions. Future research could perform the applied diagnostic assessments for the bivariate logit model on a wider and larger dataset of the two diseases.

## Introduction

Diabetes and hypertension conditions often occur concurrently in a patient^1–3^. This is usually the case because the two disease conditions share risk factors, which among others include old age, obesity, genetic factors, and dyslipidaemia^3^. Living with the two disease conditions generally affects the immune system and mental health of a patient. In particular, co-existence of type2 diabetes mellitus and hypertension in a patient is associated with poor cognitive functioning, ischemic cerebrovascular disease, retinopathy, heart disease, sexual inactivity, and kidney problems^4–7^. For patients with other chronic diseases such as cancer, presence of the diabetes and hypertension diseases is associated with poor prognosis outcomes in the patient^8–10^. Therefore, more ways of managing effects of the joint occurrence of diabetes and hypertension in cancer patients have to be explored^11^.

Since the data for observing presence or absence of either diabetes or hypertension in a cancer patient are binary, assessment of risk factors for the comorbidity of the two diseases can be done using bivariate logistic regression, a model that quantifies effects of the covariates on the joint binary outcomes, while accounting for the correlation of the two binary outcome variables^12,13^. Although this method is popular for modelling comorbidity data, the follow-up residual assessments are rarely done by researchers, mainly because the diagnostic statistics for nonlinear multivariate models are not well-developed^14^. This creates a vacuum in understanding the fit of the model to the bivariate data^15^. As for cancer patients with comorbid diabetes and hypertension, there could be some outlier patients with special characteristics, whose knowledge can help in managing joint adverse effects of the two diseases in a patient. This study therefore analyses characteristics of the outlier cancer patients with comorbid diabetes and hypertension, upon fitting a bivariate logistic regression model to the cancer patients’ data, that were randomly collected at Queen Elizabeth and Kamuzu central hospitals in Malawi. An outlier observation is one whose measurement does not conform with the rest of the data points in the fitted model^15^. This may be due to natural causes, which in the case of comorbid diabetes and hypertension patient data may help in revealing ways for managing impact of presence of the two diseases in a cancer patient. At times, outliers are as a result of data collection and handling errors. Whichever is the reason for outlierness, presence of outliers in a model may lead to biased estimated effects of risk factors of the diseases and inaccurate conclusions, as well as compromised policy directions from the fitted model.

Understanding cancer and allied diseases’ management approaches is an ongoing process, that requires continuous research^11^. Knowing the characteristics of outlier cancer patients with comorbid diabetes and hypertension contributes to that process. Following this section is the presentation in “Methods” section about the data and statistical methods used in this study. Thereafter, we present results in “Results” section. The discussion and conclusion are respectively given in “Discussion” and “Conclusion” sections.

## Methods

### Data

The study used data for adult cancer patients aged 18 years and above, that were collected at the two major referral hospitals of Queen Elizabeth and Kamuzu Central in Malawi, by the cancer research group of Kamuzu University of Health Sciences. The project engaged a random cross-sectional sample of 398 cancer patients, that presented themselves for oncological services at the two referral hospitals between 13th January and 23rd March 2021. The study participants were selected using simple random sampling technique, and face-to-face interviews were conducted to collect the data. In addition, the patients’ health passports, registers, and files were reviewed to get the patients’ histories. The data included cancer diagnosis information for the patient, its stage and treatment options, as well as some known behavioural risk factors and socio-demographic characteristics of the patient. Further details about the project can be found in the article^11^.

In this present study, interest was on identifying characteristics of cancer patients that presented outlying diabetes and hypertension measurements upon considering possible risk factors of both diseases in a regression model set up. Therefore, the outcome variables for this study were whether or not a cancer patient suffered from diabetes or hypertension or both, as at the time of the survey. The two disease conditions were selected for study as they are known to occur jointly in a patient in most cases^3^. Some of the factors that are associated with comorbid diabetes and hypertension are old age, elevated cholesterol, cigarette smoking, physical activity, wealth index, occupation, alcohol consumption status, marital status, place of residence, education level, and sex of an individual^1,16,17^. This study included these factors during the model fitting. The research team in this project got the ethical clearance and approval from the College of Medicine Research and Ethics Committee (COMREC), certificate number P.07/20/3085. In addition, the directors at the two referral hospitals provided separate approvals to allow the team to collect the data. During data collection and analysis, patients’ identities were anonymised to maximise the patients’ confidentiality and respect their privacy. In addition, informed consent to participate in this project was obtained from all participants and/or their legal guardian(s). All methods in this study were performed in accordance with the relevant guidelines and regulations^11^.

### Bivariate logistic regression model and its estimation

Let $(Y_{i1},Y_{i2})$ be a pair of jointly occurring total number of diabetes and hypertension cases observed at a health facility during some period of time, with respective paired measurements $(y_{i1}, y_{i2})$, where $i = 1, 2, ..., n$. Let 1 denotes presence of either disease in a patient and 0 absence of the disease. Then, in one such observation, the following ordered pairs are the possible outcomes: $(y_{i1},y_{i2})=\{(0,0),(0,1),(1,0),(1,1)\}$, with respective joint probabilities of occurrence denoted by: $p_{00}=P(Y_{i1}=0,Y_{i2}=0)$, $p_{01}=P(Y_{i1}=0,Y_{i2}=1)$, $p_{10}=P(Y_{i1}=1,Y_{i2}=0)$, and $p_{11}=P(Y_{i1}=1,Y_{i2}=1)$, in which $p_{00}+p_{10}+p_{01}+p_{11}=1$. If $\theta _{1}=P(Y_{i1}=1)$ and $\theta _{2}=P(Y_{i2}=1)$ are marginal probabilities of presence of the respective disease outcomes in one observation, and $\sigma _{12}=Cov(Y_{i1},Y_{i2})$ is the covariance of the two disease outcomes, then $(Y_{i1},Y_{i2})$ has a bivariate binomial distribution in *n* observations, expressed as follows:

1 $$\begin{aligned} \begin{bmatrix} Y_{i1}\\ Y_{i2} \end{bmatrix} \sim Binomial_{2}\left( \begin{bmatrix} n_{1}\\ n_{2} \end{bmatrix}, \begin{bmatrix} \theta _{1}\\ \theta _{2} \end{bmatrix} \right) , \end{aligned}$$

where $n_{1}=n_{2}=n$ is the number of times the experiment was jointly performed to observe the two outcomes. Therefore, the probability mass function (pmf) of the pair $(Y_{i1},Y_{i2})$, expressed as $P((Y_{i1}=y_{i1},Y_{i2}=y_{i2})|n,\theta _{1},\theta _{2})=h(y_{i1},y_{i2}|n,\theta _{1},\theta _{2})$ is given by:

2 $$\begin{aligned} \begin{aligned} h(y_{i1},y_{i2}|n,\theta _{1},\theta _{2})&=\frac{n!}{y_{i1}!y_{i2}!(n-y_{i1}-y_{i2})!}\theta _{1}^{y_{i1}}\theta _{2}^{y_{i2}}(1-\theta _{1}-\theta _{2})^{n-y_{i1}-y_{i2}}\\&=\exp \left[ y_{i1}\log \frac{\theta _{1}}{1-(\theta _{1}+\theta _{2})}+y_{i2}\log \frac{\theta _{2}}{1-(\theta _{1}+\theta _{2})}+n\log (1-(\theta _{1}+\theta _{2}))+\log (A)\right] , \end{aligned} \end{aligned}$$

where $A=\frac{n!}{y_{i1}!y_{i2}!(n-y_{i1}-y_{i2})!}$^18^. As it can be appreciated in Eq. (2), the bivariate binomial distribution is in canonical form, and two natural parameters come out clearly, these are: $\log \frac{\theta _{1}}{1-(\theta _{1}+\theta _{2})}$ and $\log \frac{\theta _{2}}{1-(\theta _{1}+\theta _{2})}$. Hence, a simultaneous logistic regression model will have to be defined in terms of these two link functions and solved simultaneously to find effects of covariates on the bivariate response $(Y_{i1},Y_{i2})$.

Let ${\textbf {x}}^{T}_{ik}=(1,x_{i1},x_{i2},...,x_{ip})$ be a vector of explanatory variables’ values observed on the *i*-th patient of both diabetes and hypertension, where $x_{i0}=1$. Then, the following expression characterises the bivariate logistic regression model:

3 $$\begin{aligned} \begin{aligned} Y_{ij}&=\theta _{ij}({\textbf {x}}) + \epsilon _{ij}, i = 1,2,...,n; j=1,2,\\ \sigma _{12}&=g({\textbf {x}}), \end{aligned} \end{aligned}$$

where $Y_{ij}$ is the bivariate binary response variable, i.e. $(Y_{i1}, Y_{i2})$; $\theta _{ij}({\textbf {x}})$ is the marginal probability of success for $Y_{i1}$ or $Y_{i2}$ given covariates, with $0 \le \theta _{ij}({\textbf {x}})\le 1$; $\sigma _{12}$ is the covariance term capturing dependence between $Y_{i1}$ and $Y_{i2}$, which is a function of some covariates ${\textbf {x}}$, usually estimated by the odds ratio $\frac{p_{00}p_{11}}{p_{01}p_{10}}$; and $\epsilon _{ij}$ is the model’s error term^19^. Assuming $\epsilon _{ij}$ has mean zero, then the conditional expectation of the response $Y_{ij}$ given covariates, i.e. $E(Y_{ij}|X)$ is a function of marginal probabilities of success only, $\theta _{ij}({\textbf {x}})$, which is the part that relates or links the model with the covariates^19^. This leads to the explicit definition of the bivariate logit model in Eq. (3) through the link functions in Eq. (2), given by:

4 $$\begin{aligned} \begin{aligned} \log \frac{\theta _{i1}({\textbf {x}})}{1-(\theta _{i1}({\textbf {x}})+\theta _{i2}({\textbf {x}}))}&={\textbf {x}}_{1ik}^{T}\beta , \\ \log \frac{\theta _{i2}({\textbf {x}})}{1-(\theta _{i1}({\textbf {x}})+\theta _{i2}({\textbf {x}}))}&={\textbf {x}}_{2ik}^{T}\beta ,\\ \log \left[ \frac{p_{00}p_{11}}{p_{01}p_{10}}\right]&={\textbf {x}}_{3ik}^{T}\beta , \end{aligned} \end{aligned}$$

where $\beta ^{T}=(\beta _0,\beta _1,...,\beta _p)$ is a vector of model parameters and ${\textbf {x}}_{ik}=(1,x_{i1},x_{i2},...,x_{ip})^{T}$ is a vector of explanatory variables measured on *i*-th patient, while ${\textbf {x}}_{1ik}^{T}\beta$, ${\textbf {x}}_{2ik}^{T}\beta$, and ${\textbf {x}}_{3ik}^{T}\beta$ are the model’s linear operators, respectively, associated with first marginal model, the second marginal model, and the covariance model for odds ratio. In this scenario, the effects of covariates on the marginal outcomes and covariance term are said to be non-exchangeable or parallel. Alternatively, the same covariates may be used for the tripartite model in Eq. (4), in which case the effects of covariates on the marginal outcomes and covariance term are said to be exchangeable, where the same fixed-effects apply to both marginal outcomes and the covariance term. In this study, both non-exchangeable (parallel) and exchangeable types of the bivariate logit model were fitted to the data. The two outcome variables, $Y_{i1}$ and $Y_{i2}$ are independent if and only if $\log \big [\frac{p_{00}p_{11}}{p_{01}p_{10}}\big ]=0$ or the odds ratio $\frac{p_{00}p_{11}}{p_{01}p_{10}}=1$. Through algebra, one can derive an alternative form of the bivariate logistic regression model in Eq. (4) in terms of the two marginal probabilities of the success for the two response variables, i.e., $\theta _{i1}({\textbf {x}})$ and $\theta _{i2}({\textbf {x}})$, and the odds ratio term, $\frac{p_{00}p_{11}}{p_{01}p_{10}}$ as follows:

5 $$\begin{aligned} \begin{aligned} \theta _{i1}({\textbf {x}})&=\frac{\exp ({\textbf {x}}_{1ik}^{T}\beta )}{1+\exp ({\textbf {x}}_{1ik}^{T}\beta )+\exp ({\textbf {x}}_{2ik}^{T}\beta )}, \\ \theta _{i2}({\textbf {x}})&=\frac{\exp ({\textbf {x}}_{2ik}^{T}\beta )}{1+\exp ({\textbf {x}}_{1ik}^{T}\beta )+\exp ({\textbf {x}}_{2ik}^{T}\beta )},\\ \frac{p_{00}p_{11}}{p_{01}p_{10}}&=\exp ({\textbf {x}}_{3ik}^{T}\beta ). \end{aligned} \end{aligned}$$

The likelihood function for the bivariate logit model in Eqs. (4) or (5) is constructed by taking the product of probabilities of number of events in Eq. (2) associated with each *i*-th observation as follows:

6 $$\begin{aligned} \small L(\theta )=\prod _{i=1}^{n}\exp \left[ y_{i1}\log \frac{\theta _{i1}({\textbf {x}})}{1-(\theta _{i1}({\textbf {x}})+\theta _{i2}({\textbf {x}}))}+y_{i2}\log \frac{\theta _{i2}({\textbf {x}})}{1-(\theta _{i1}({\textbf {x}})+\theta _{i2}({\textbf {x}}))}+n\log (1-(\theta _{i1}({\textbf {x}})+\theta _{i2}({\textbf {x}})))+\log (A)\right] , \end{aligned}$$

where $A=\frac{n!}{y_{i1}!y_{i2}!(n-y_{i1}-y_{i2})!}$. Taking partial derivatives of the log-likelihood function with respect to the model parameters will give the score vectors, which upon equating to zero and solving for the parameters we can obtain the maximum likelihood (ML) estimates for the regression coefficients, $\beta$. The exponentiated fixed effect ML estimates, $exp({\hat{\beta }})$ have usual interpretation as marginal odds ratios of success in the respective outcome, when comparing one level of a covariate to the other called reference level. The covariance term was estimated as in third line of the model in Eq. (5), and it was interpreted as the degree of dependence between the two response variables $Y_{i1}$ and $Y_{i2}$. A positive logarithm of odds ratio implied that the first disease outcome was highly likely to occur in a person than the second disease, while a negative value meant the first disease was less likely to occur than the second. The value of zero meant that there was no association between the two disease outcomes. The model estimates were calculated using the R package VGAM, that is used to fit vector generalised linear and additive models^20^, while the rest computations of the residuals and graphs were also performed in R software version 4.2.2 using appropriate packages.

Before analysing the outlier observations to the fitted model, the available explanatory variables were reviewed, so that only those that resulted into a better model according to the deviance statistic, i.e. $D(y_{ij},{\hat{\theta }})=\sum _{i=1}^{n}\sum _{j=1}^{2}2\times [ \log L({\hat{\theta }}_{sj};y_{ij})-\log L({\hat{\theta }}_{0j};y_{ij})]$ could be involved in the final analyses, where ${\hat{\theta }}_{0j}$ stands for ML estimates in reduced model, ${\hat{\theta }}_{sj}$ the estimates in saturated or full model, and $\log L({\hat{\theta }})$ an estimate of the log-likelihood. The first model had all available covariates as given in “Data” section. While, in the second model few were dropped based on large p-values. The larger the deviance statistic’s value the better the fit of the model to the data.

### Model’s outlier residual analysis

To analyse joint outlier observations to the model in Eqs. (3)–(5), we first defined a deviance residual for tracking outliers to the marginal models in the bivariate logit model as follows:

7 $$\begin{aligned} \small d_{ij} = sgn(y_{ij}-{\hat{\theta }}_{ij}({\textbf {x}})) \left[ -2[y_{ij}\log {\hat{\theta }}_{ij}({\textbf {x}})+(1-y_{ij})\log(1-{\hat{\theta }}_{ij}({\textbf {x}}))]\right] ^{1/2}, \end{aligned}$$

where $y_{ij}$ is *i*-th observation for the *j*-th response, ${\hat{\theta }}_{ij}({\textbf {x}})=\frac{exp({\textbf {x}}_{jik}^{T}{\hat{\beta }})}{1+exp({\textbf {x}}_{1ik}^{T}{\hat{\beta }})+exp({\textbf {x}}_{2ik}^{T}{\hat{\beta }})}$ is the fitted marginal probability of event in *j*-th outcome for the *i*-th subject, *sgn*(.) is the signum function of the residual $y_{ij}-{\hat{\theta }}_{ij}({\textbf {x}})$, which was $+1$ if the residual was above zero, $-1$ when the residual was negative, and 0 if the residual was zero, $i=1,2,...,n$, and $j=1,2$. The deviance residual in Eq. (7) is assumed to follow a normal distribution, such that its extreme values will correspond to outlier observations to the fitted marginal model^19^. We assessed this graphically by plotting the marginal deviance residuals in Eq. (7) against fitted marginal probabilities of success, ${\hat{\theta }}_{ij}({\textbf {x}})$. The overall joint outlier observations to the entire bivariate logit model were analysed by taking the average of the marginal deviance residuals in Eq. (7), as though each column of the bivariate binary outcome was a level in a multinomial random variable^21–23^. Therefore, the statistic for assessing these overall outliers is defined as follows:

8 $$\begin{aligned} D_{i}=\frac{1}{2}(d_{i1}+d_{i2}), \end{aligned}$$

where $d_{i1}$ and $d_{i2}$ are respective marginal deviance residuals obtained in Eq. (7). Large absolute values of the overall deviance residual in Eq. (8) correspond to joint outlier observations to the fitted bivariate logit model. Graphical aids were also used for this analysis, by plotting the residual in Eq. (8) against fitted marginal probabilities of success, ${\hat{\theta }}_{ij}({\textbf {x}})$, as well as against marginal deviance residuals in Eq. (7). Upon identifying outlier observations to the fitted bivariate logit model, we performed back-inspection in the dataset to trace the characteristics of the identified outlier patients.

### Ethical approval and consent to participate

The study was part of the Kamuzu University of Health Sciences cancer project, whose data were collected with approval of the College of Medicine Research and Ethics Committee (COMREC). The ethical approval certificate is numbered P.07/20/3085. Subsequent approvals were also given by the respective directors at Queen Elizabeth and Kamuzu Central hospitals. The three approval letters have been submitted together with this manuscript. All ethical considerations for the patients were adhered to during data collection, analysis and reporting of this study. Informed consent to participate in this project was obtained from all participants and/or their legal guardian(s). All methods in this study were performed in accordance with the relevant guidelines and regulations. More details on ethical clearance for the project are provided in the article^11^.

## Results

### Bivariate logit model estimates

The summary of the data is given in Table 1. It was shown that cases of diabetes and hypertension were respectively present in 0.51% and 8.10% of the studied cancer patients. The diabetes cases were much concentrated in female, unmarried, employed or retired, rural-based, non-smoking, and non-alcohol drinking, above 30 age groups, and patients with primary education and above. While hypertension cases were more dominant in female, married, unemployed or students, urban, non-alcohol drinking, and above 30 age groups. The chi-square test of association indicated that there was evidence from the data that age category of a person was associated with hypertension (p-value = 0.030). While sex of a person had marginal association with hypertension (p-value = 0.083). There was no evidence of association, at 5% significance level, between each studied variable and diabetes.

**Table 1:** Distribution of diabetes and hypertension cases by socio-demographic characteristics of a patient.

Characteristic	n (%)	Diabetes		Hypertension	
		Cases (%)	$\chi ^{2}$ p-value	Cases (%)	$\chi ^{2}$ p-value
Overall sample	395 (100)	2 (0.51)		32 (8.10)	
Sex			0.288		0.083
Male	142 (35.95)	0 (0.00)		7 (4.93)	
Female	253 (64.05)	2 (0.79)		25 (9.88)	
Marital status			0.636		0.451
Unmarried	135 (34.18)	1 (0.74)		9 (6.67)	
Married	260 (65.82)	1 (0.38)		23 (8.85)	
Occupation			0.173		0.543
Unemployed/student	189 (47.85)	0 (0.00)		17 (8.99)	
Employed/retired	205 (51.90)	2 (0.98)		15 (7.32)	
Residential place			0.259		0.552
Urban	153 (38.73)	0 (0.00)		14 (9.15)	
Rural	241 (61.01)	2 (0.83)		18 (7.47)	
Highest education			0.495		0.996
None	74 (18.73)	0 (0.00)		6 (8.11)	
Primary and above	320 (81.01)	2 (0.63)		26 (8.13)	
Age category			0.602		0.030
18–30	47 (11.90)	0 (0.00)		0 (0.00)	
31+	348 (88.10)	2 (0.57)		32 (9.20)	
Ever smoked cigarette			0.585		0.998
No	337 (85.32)	2 (0.59)		27 (8.01)	
Yes	50 (12.66)	0 (0.00)		4 (8.00)	
Ever drank alcohol			0.496		0.636
No	312 (78.99)	2 (0.64)		27 (8.65)	
Yes	72 (18.23)	0 (0.00)		5 (6.94)	

The estimates from a bivariate logit model with non-exchangeable covariates effects on diabetes and hypertension outcomes are given in Table 2. Model 2, that excluded occupation and age variables, had a higher value of deviance statistic compared to Model 1, hence it relatively had a better fit, and was used for subsequent outlier analyses. The intercept estimates in Model 2 showed that, without considering the covariates, the logarithm of odds of suffering from diabetes or hypertension was lower in the studied population compared to not suffering from either condition. While the intercept of the covariance term was larger than 0, indicating that, holding the factors constant, suffering from diabetes was more likely than hypertension. The marginal ML estimates in Model 2 showed that, adjusting for the other factors, the logarithm of odds of suffering from either diabetes or hypertension was higher in female compared to male persons, in the educated compared to uneducated, and in persons with alcohol drinking history. Whereas, log-odds of diabetes was lower in married persons, but for hypertension it was higher in the married compared to unmarried persons. Diabetes chances were also low in persons with history of smoking, but for hypertension disease they were high in the persons who ever smoked tobacco compared to those who never smoked. Finally, the results showed that diabetes was more likely in rural, but hypertension was less likely to rural residents compared to urban dwellers. The p-values for the estimates in the non-exchangeable bivariate logit model in Model 2 were still large even after dropping occupation and age group variables, and some did not process.

**Table 2:** Effect of patient characteristics on diabetes and hypertension outcomes upon fitting bivariate logit model to the data, with non-exchangeable effects.

Variable	Model 1			Model 2		
	Diabetes	Hypertension	Covariance	Diabetes	Hypertension	Covariance
	Log-odds (p-val)	Log-odds (p-val)	Log-OR (p-val)	Log-odds (p-val)	Log-odds (p-val)	Log-OR (p-val)
Intercept	− 547.037 (0.996)	− 272.044 (0.998)	9.165 (1.000)	− 28.632 (NA)	− 3.454 (0.895)	6.377 (NA)
Sex						
Male*						
Female	5.513 (0.728)	0.799 (0.449)	6.165 (NA)	6.506 (0.808)	0.937 (0.488)	9.867 (0.958)
Marital status						
Unmarried*						
Married	− 0.444 (0.748)	0.308 (0.752)	13.603 (NA)	− 0.541 (0.704)	0.387 (0.592)	17.347 (0.903)
Occupation						
Unemployed/student*						
Employed/retired	9.492 (0.945)	− 0.248 (0.939)	1.294 (0.998)			
Residential place						
Urban*						
Rural	9.289 (0.955)	− 0.305 (0.848)	9.453 (0.969)	9.211 (0.953)	− 0.320 (0.906)	− 8.026 (0.982)
Highest education						
None*						
Primary and above	1.418 (0.660)	0.419 (0.807)	− 12.433 (0.845)	9.230 (0.998)	0.265 (0.992)	− 15.495 (0.993)
Age category						
18–30*						
31+	518.411 (0.996)	268.885 (0.998)	− 16.885 (1.000)			
Ever smoked cigarette						
No*						
Yes	0.379 (0.956)	0.351 (0.882)	6.871 (0.970)	− 2.959 (NA)	0.348 (0.860)	13.021 (NA)
Ever drank alcohol						
No*						
Yes	1.932 (0.913)	0.103 (0.945)	3.894 (NA)	2.932 (0.906)	0.165 (0.925)	7.184 (0.973)
Deviance	213.4181			224.9023		

*OR* odd ratio, *NA* not applicable to base variable selection on, estimate was affected by the Hauck–Donner effect^24^, see “Discussion” section for details.

*Reference level.

The results in Table 3 are for the case of exchangeable covariates effects on diabetes and hypertension in the fitted bivariate logit model. Model 4, that excluded occupation and age group had a better fit, based on the deviance statistic value. The ML estimates for the intercept showed that, disregarding the covariates, a person was less likely to suffer from diabetes or hypertension compared to not suffering from either disease. The covariance estimate showed that, holding the covariates constant, diabetes event was less likely to occur in the study population compared to hypertension. This agreed with the summary data in Table 1. The marginal ML estimates showed that suffering from diabetes or hypertension was more likely in females compared to males, in married compared to unmarried, in persons with primary education and above compared to the uneducated, and in persons with history of smoking or alcohol drinking. The two diseases were less likely to occur in persons that resided in rural areas. The p-values of estimates in the exchangeable bivariate logit model in Table 3 were generally lower compared to those of non-exchangeable model estimates in Table 2, and the model overall deviance statistics values were higher than in non-exchangeable model. This indicated that the exchangeable model fitted the data better.

**Table 3:** Effect of patient characteristics on diabetes and hypertension outcomes upon fitting bivariate logit model to the data, with exchangeable effects.

Variable	Model 3			Model 4		
	Diabetes	Hypertension	Covariance	Diabetes	Hypertension	Covariance
	Log-odds (p-val)	Log-odds (p-val)	Log-OR (p-val)	Log-odds (p-val)	Log-odds (p-val)	Log-OR (p-val)
Intercept	− 503.552 (0.995)	− 503.552 (0.995)	2.557 (1.000)	− 4.344 (NA)	− 4.344 (NA)	− 54.216 (NA)
Sex						
Male*						
Female	0.878 (0.202)	0.878 (0.202)	3.054 (0.583)	0.967 (0.141)	0.967 (0.141)	5.809 (0.768)
Marital status						
Unmarried*						
Married	0.258 (0.682)	0.258 (0.682)	5.919 (0.474)	0.336 (0.533)	0.336 (0.533)	9.663 (0.669)
Occupation						
Unemployed/student*						
Employed/retired	0.042 (0.990)	0.042 (0.990)	32.753 (0.999)			
Residential place						
Urban*						
Rural	− 0.111 (0.838)	− 0.111 (0.838)	11.456 (0.840)	− 0.129 (0.776)	− 0.129 (0.776)	8.857 (0.654)
Highest education						
None*						
Primary and above	0.323 (0.734)	0.323 (0.734)	− 7.054 (NA)	0.411 (0.971)	0.411 (0.971)	31.799 (1.000)
Age category						
18–30*						
31+	499.532 (0.995)	499.532 (0.995)	− 45.182 (1.000)			
Ever smoked cigarette						
No*						
Yes	0.339 (0.731)	0.339 (0.731)	0.260 (0.969)	0.289 (0.712)	0.289 (0.712)	− 1.0181 (0.884)
Ever drank alcohol						
No*						
Yes	0.159 (0.867)	0.159 (0.867)	4.116 (0.570)	0.294 (0.720)	0.294 (0.720)	9.700 (0.628)
Deviance	250.9608			262.4044		

*OR* odd ratio, *NA* not applicable to base variable selection on, estimate was affected by the Hauck–Donner effect^24^, see “Discussion” section for details.

*Reference level.

### Outlier cancer patients and their characteristics

**Figure 1:**
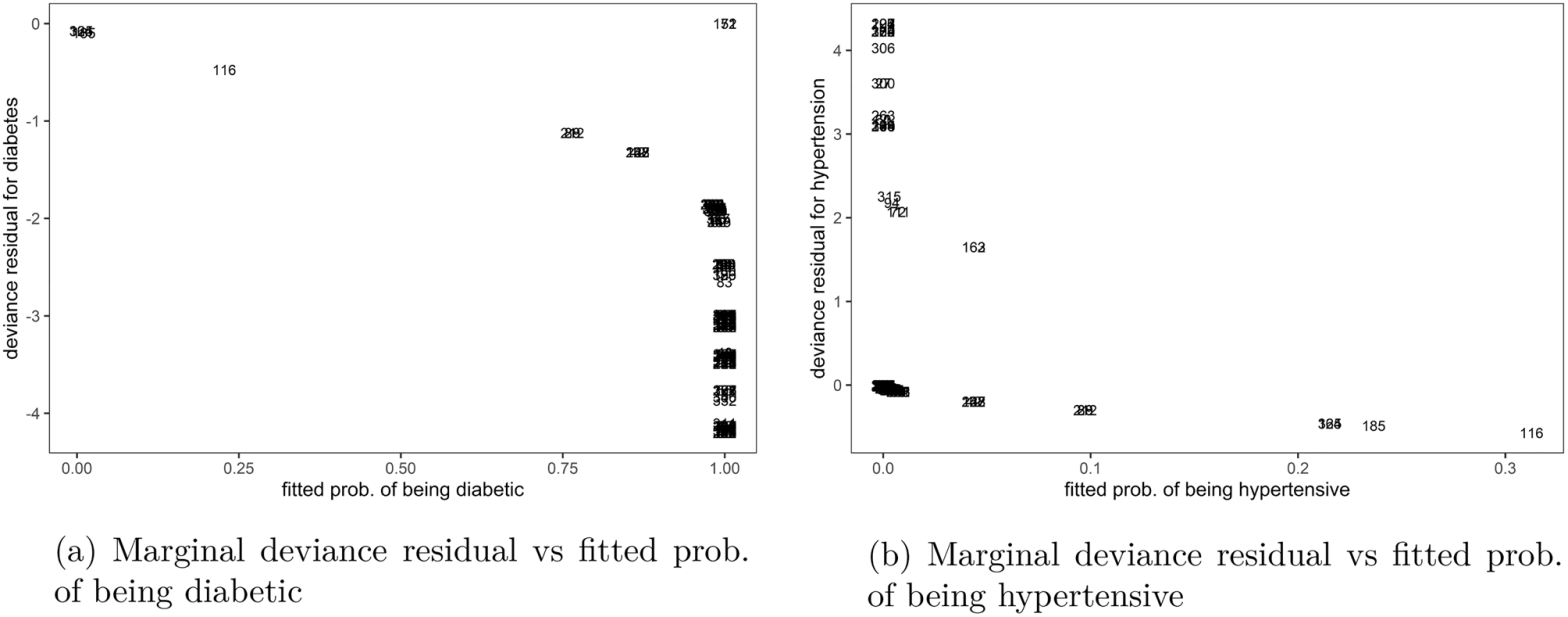
Outliers to marginal outcomes in non-exchangeable bivariate logit model, Malawi cancer patients data. *Source* Researcher.

The plots of marginal deviance residuals against respective fitted probabilities in Fig. 1 showed that the non-exchangeable bivariate logit model was poorly fitted to the data. Using a cutoff of $\pm \, 2.5$, most residual values for analysing the fit of subjects to diabetes outcomes in Fig. 1a were outside this margin, which was also the case with those for assessing the fit to hypertension outcomes in Fig. 1b. The data also showed that the diabetes model over-predicted majority of the observations, i.e. their real measurements were lower than those estimated by the model, see Fig. 1a. While with hypertension marginal model, the majority of the observations were under-predicted by the model as in Fig. 1b, suggesting that their real values were higher than those estimated by the model.

**Figure 2:**
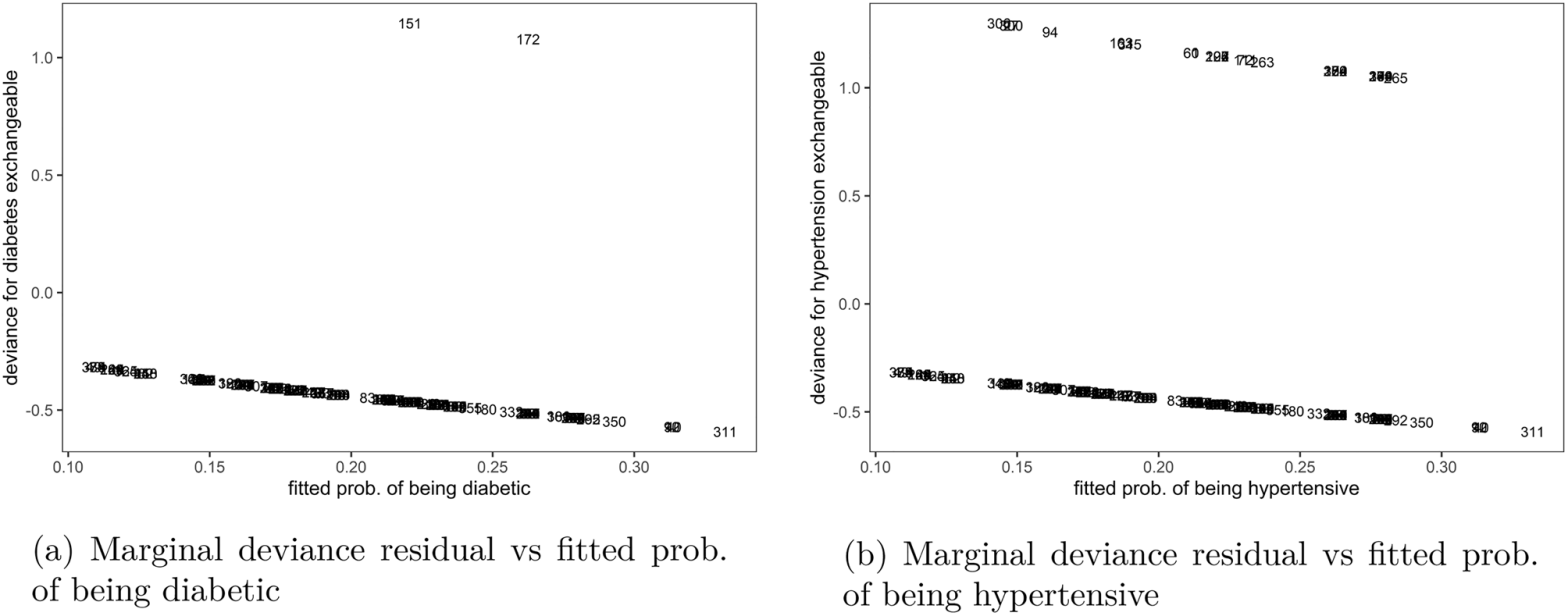
Outliers to marginal outcomes in exchangeable bivariate logit model, Malawi cancer patients data. *Source* Researcher.

Unlike with the non-exchangeable marginal models, the residual results for exchangeable marginal models in Fig. 2 showed that majority of the observations were well-fitted by the model. Most estimates for the fit of observations to diabetes marginal outcomes in Fig. 2a or hypertension outcomes in Fig. 2b were close to the zero line. It was further shown that at a cutoff of $\pm \, 1$ few observations in both marginal models, such as patients number 151 and 172 were under-predicted by the model, as their plots were outside these margins, indicating that these were possible candidates for outliers.

**Figure 3:**
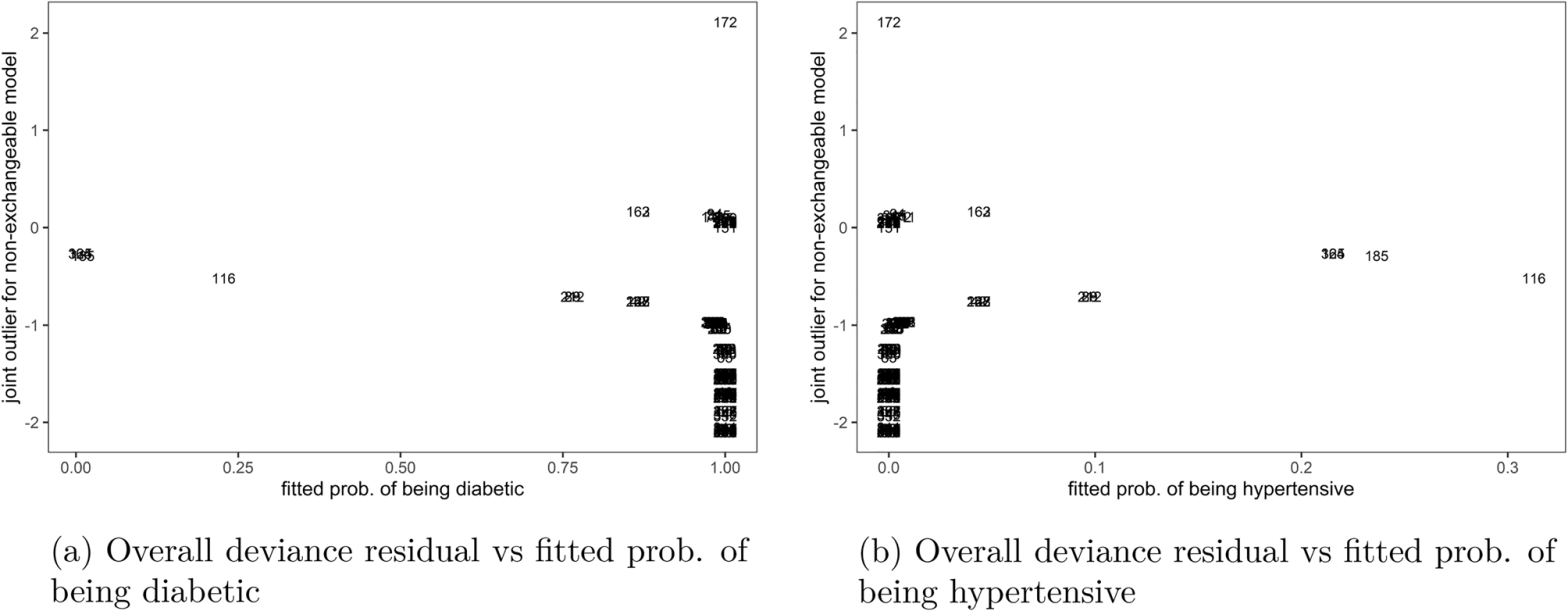
Joint outliers to the non-exchangeable bivariate logit model, Malawi cancer patients data. *Source* Researcher.

The plots of overall deviance residual against marginal fitted probabilities for non-exchangeable bivariate logit model in Fig. 3 showed that the observation with identity number 172 was an outlier to the model, on the under-predicted (positive) side, while several others were also outliers but on the over-predicted (negative) side using a cutoff of $\pm \, 2$, when the overall residual was plotted against marginal probability of diabetes, Fig. 3a or marginal probability of hypertension, Fig. 3b. This meant that, although the non-exchangeable model had poor fit as reported in previous paragraph, the applied method still detected few outlier observations to the model like number 172, that were not conforming to the pattern of the other data points in the model.

**Figure 4:**
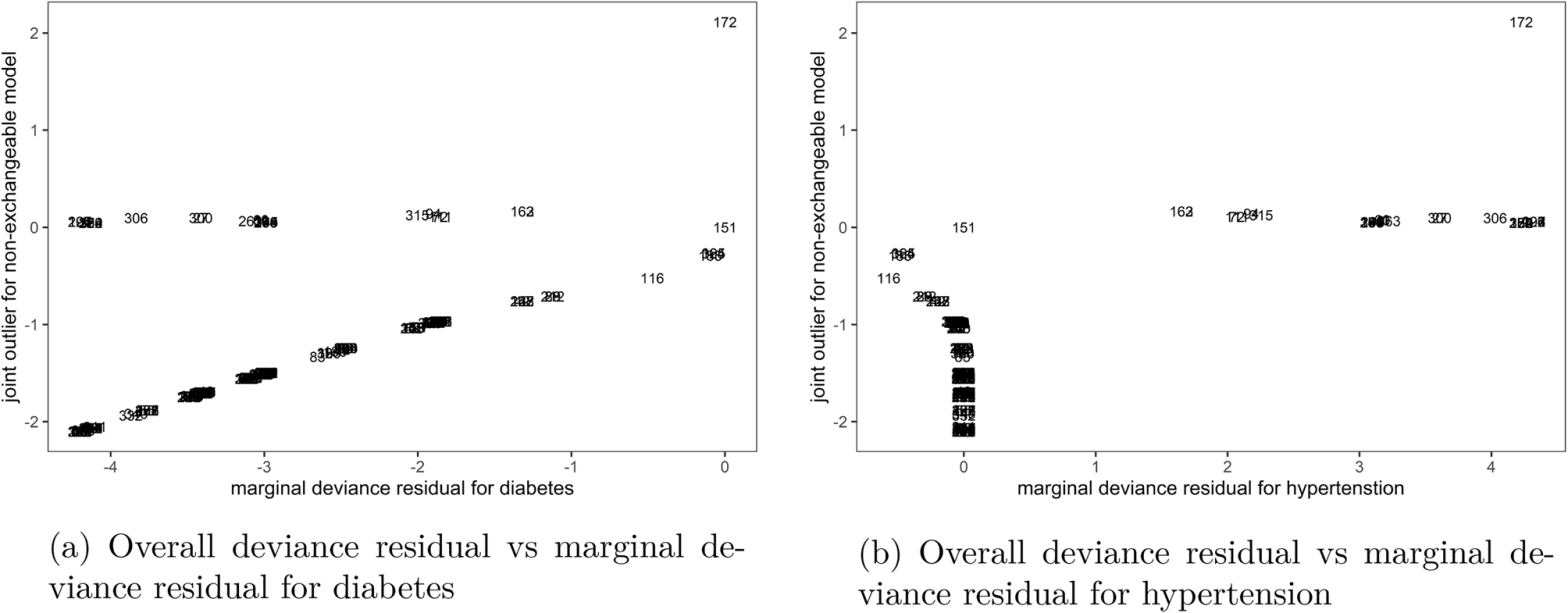
Joint outliers to the non-exchangeable bivariate logit model, Malawi cancer patients data. *Source* Researcher.

Similarly, when the overall deviance residual for the non-exchangeable model was plotted against marginal deviance residuals in Fig. 4, it was shown that observation number 172 was an outlier on a positive side and few others on the negative side, using the same cutoff of $\pm 2$. This result was observed in both Fig. 4a that plotted overall residual against marginal deviance to diabetes and Fig. 4b against hypertension outcomes.

**Figure 5:**
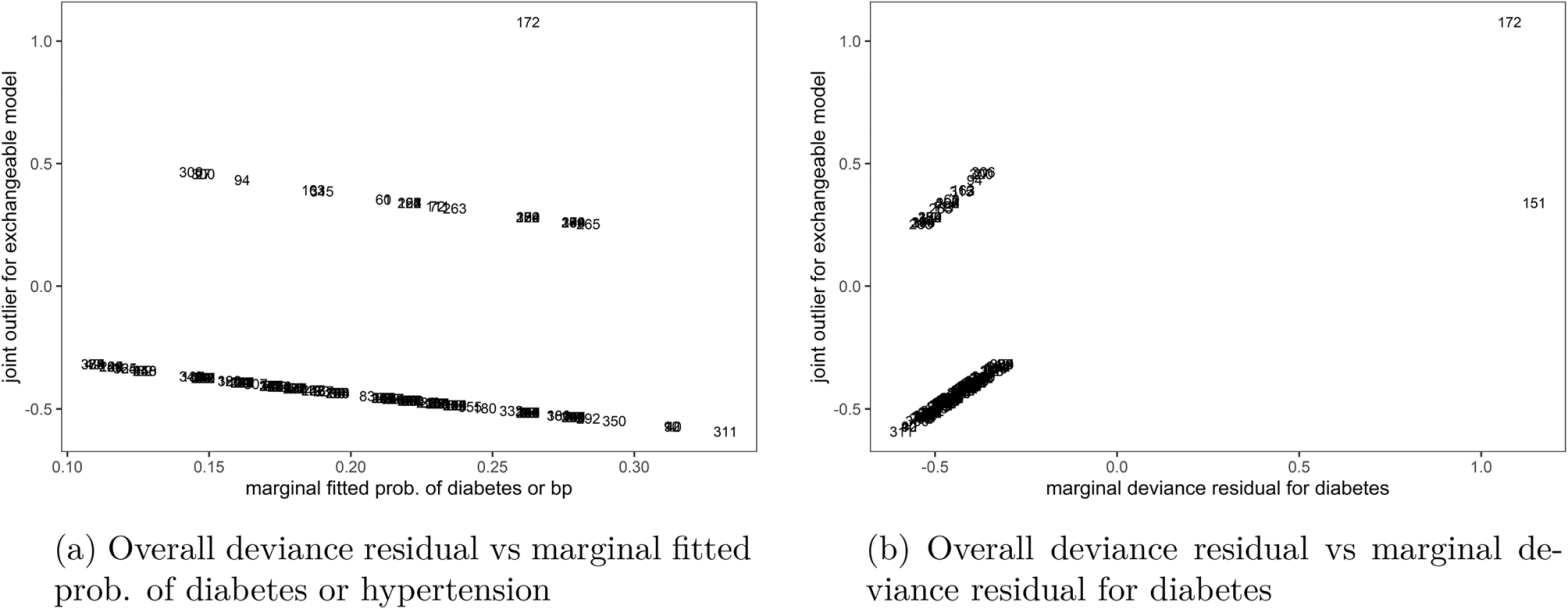
Joint outliers to the exchangeable bivariate logit model, Malawi cancer patients data. *Source* Researcher.

Now, using the cutoff $\pm \,1$ the plots of overall deviance residual for the exchangeable bivariate logit model against marginal fitted probability of being diabetic or hypertensive in Fig. 5a, as well as against marginal deviance residual for diabetes in Fig. 5b identified observation number 172 as an outright outlier to the bivariate logit model. This observation was under-predicted by the model, i.e. its real outcome measurement was higher than what was predicted by the model. The rest of the plots spanned the zero line, indicating that their corresponding observations were well-fitted by the exchangeable bivariate logit model.

**Figure 6:**
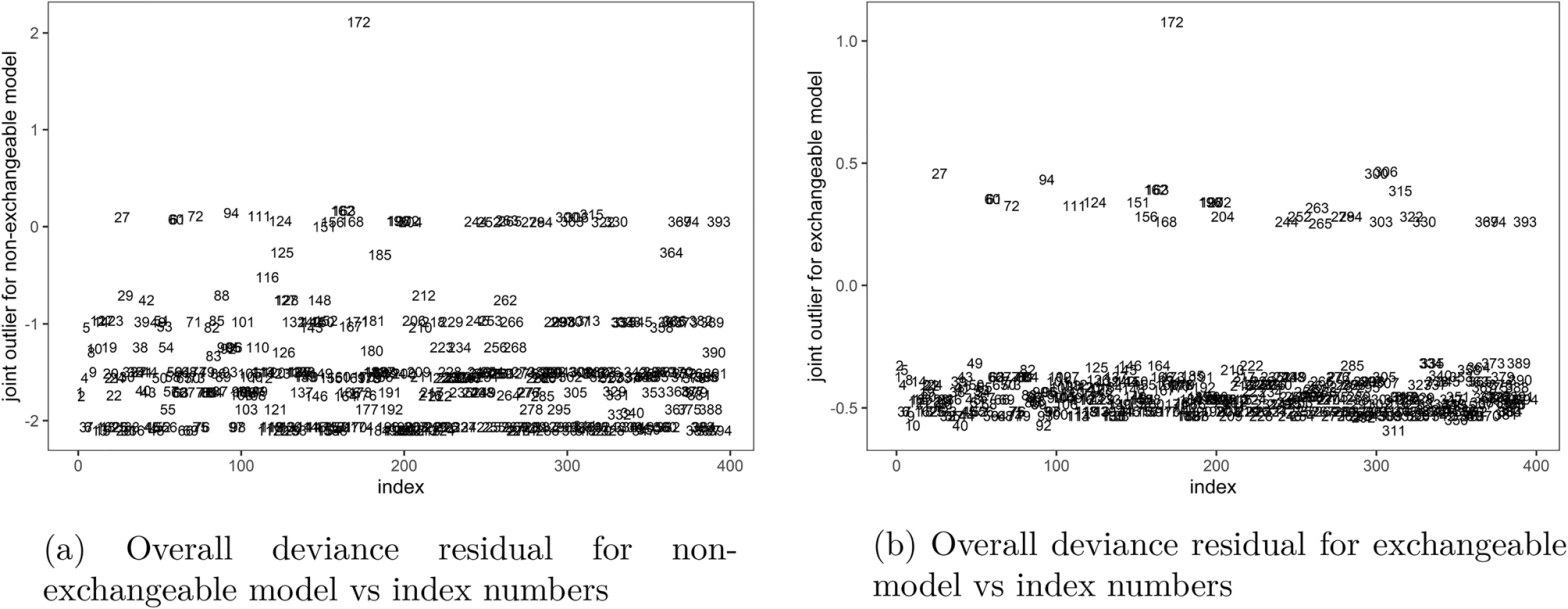
Joint outliers to the non-exchangeable and exchangeable bivariate logit models, Malawi cancer patients data. *Source* Researcher.

Finally, index plots of the overall residual in both non-exchangeable, Fig. 6a and exchangeable, Fig. 6b models showed that observation number 172 was an outlier to both models. This was the only outlier detected by the residual to the exchangeable model in Fig. 6b at cutoff $\pm \, 1$, indicating that the exchangeable model fitted the data well. However, the residual plots for the non-exchangeable model in Fig. 6a identified several other outliers at cutoff $\pm \, 2$, apart from observation number 172, indicating poor fit of the non-exchangeable model to the data compared to the exchangeable model.

Upon tracking the identified overall outlier observation number 172 in the main dataset, the results showed that this was a female aged 43 years, who weighed 61 kgs and resided in rural area in *Chikwawa* district. The outlier patient was a married Christian, with middle wealth index, minimum of secondary education, and was a retired employee as at survey time. She suffered from cervical cancer, that was diagnosed in 2018 as a localised disease. She had since received chemotherapy treatment and underwent surgery. The outlier cancer patient suffered from both diabetes and hypertension diseases, as well as HIV/AIDS, but had no any heart, liver, nor respiratory related complications. She also had no history of tobacco smoking nor alcohol drinking. The data showed that she had problems with walking about, felt moderate pain and discomforts, and had problems with performing usual activities, such as household chores or study.

## Discussion

In this article, the postestimation techniques for the bivariate logistic regression model were applied to analyse outlier cancer patients that had comorbidities of diabetes and hypertension at Queen Elizabeth and Kamuzu central hospitals in Malawi. The study identified one overall outlier patient to the two diseases’ model. The detected outlier patient was a female with cervical cancer and based in rural area, who suffered from both diabetes and hypertension. She was married and educated, with age of 43 years and moderate weight of 61 kgs. The outlier patient had no smoking and alcohol drinking history. Since this study observed that co-occurrence of diabetes and hypertension was rare for rural populations, it was therefore not surprising for the bivariate logit model diagnostic statistics to detect this rural resident as an outlier to the model^7^. In most cases, the next step after identifying outliers is to assess their influence on the model’s estimates, which is usually done by re-fitting the model to data without the outlier observations in the sample and observe the changes in the ML estimates or by applying appropriate influence statistics to examine influence of each observation on ML estimates^14,19,25^. We did not perform such analysis in this study, as the goal of the paper was on detecting and understanding characteristics of the outlier cancer patients to comorbidities of diabetes and hypertension, so as to establish the reasons for outlierness in order to recommend appropriate ways for managing the two diseases in such type of patients, without necessarily improving the model.

This study has observed that the exchangeable bivariate logit model fitted the data better than the non-exchangeable model. The data provided evidence of dependence between diabetes and hypertension diseases in a person. Based on the exchangeable model, suffering from diabetes was less likely to happen compared to hypertension in a patient. This may reflect the nature of the sample that was used, which had too few diabetes cases. The Wald test for evaluating significance of each covariate in the fitted bivariate logit model suffered from the Hauck–Donner effect (HDE) in this study, in which the test value tends to zero when the distance between the ML estimate and hypothesised value widens up, which rendered the p-values for the ML estimates to be unrealistically larger than expected or to be not applicable for variable selection analysis^24,26^. This could be as a result of smallness in sample size that was used. The HDE usually masks the true effect of a covariate on a binary outcome as though it does not exist, and this can be corrected by using alternative tests for assessing usefulness of covariates in a logit model, such as likelihood-ratio test instead of the Wald test^24^. The likelihood ratio test analyses usefulness of a covariate by comparing the likelihood estimates with and without the particular covariate in the model. However, this method requires re-fitting the model to data upon removing the concerned covariate, which can be cumbersome for non-linear models, such as the bivariate logistic regression model, that engage iterative numerical techniques like Newton–Raphson method to estimate parameters^27^. Hence, this study used the Wald test to analyse significance of covariates, due to its computation efficiency. Although the p-values realised from the models were large, this study proceeded with joint outlier analysis to the bivariate logit model because outlierness of an observation is relative to others in the fitted model^25^, no matter the level of fit of the model at hand.

The small sample size used in this study might have also affected Chi-Square test results in the cross-classification analysis, where some variables were observed to have insignificant association at 5% significance level with either diabetes or hypertension. However, the Chi-Square p-values for the affected variables were still far lower that 1, which indicated existence of some association, at higher levels of significance than 5%, between the concerned variables and diabetes or hypertension. These results add to the debate on whether researchers should prioritise statistical significance over clinical significance for studies that involve some medical data^28–30^. The general consensus in literature is that statistical insignificance should not override clinical significance^31^. It is for this reason that this study included all the affected variables during the Chi-Square tests when fitting the bivariate logit model to the data to analyse outlier cancer patients.

Finally, the study observed that there was high likelihood for female, the married, the educated, and persons with smoking and alcohol drinking history to suffer from either diabetes or hypertension. The diseases were less likely to affect the rural compared to urban populations. These results were consistent with literature, that attributes fancy lifestyle in the indicated populations as a source for increased risk^3^.

## Conclusion

This study sought to assess joint outliers to comorbidities of diabetes and hypertension through fitting a bivariate logistic regression model to the cancer medical data in Malawi. By applying diagnostic statistics for multivariate data models, we effectively examined outliers to the fitted bivariate logit model. The methods identified one outlier cancer patient to diabetes and hypertension outcomes, whose main unique feature was being based in rural area, where the two studied diseases were rare. We recommend careful analysis of outlying patients to comorbid diabetes and hypertension, through regression methods that should precede drafting of policies for managing the two diseases in cancer patients, in order to avoid misaligned interventions.

Further, the model estimates in this study confirmed previously-observed risk factors for comorbidities of diabetes and hypertension. The most vulnerable groups were females, the educated, past smokers and drinking groups, and the married. The exchangeable bivariate logit model produced better fit compared to the non-exchangeable model. Therefore, assuming that the available covariates affect either marginal outcome in the bivariate logit model could give the researcher accurate estimates, when using limited data. There are a number of risk factors for comorbid diabetes and hypertension, that are reported in literature but were not part of the dataset used in this study, moreover the sample was small which affected convergence of estimates such as the p-values. Future research could apply these statistical methods on a wider scale dataset.

## Data Availability

The dataset that was used and analysed for this study is available from the corresponding author upon request
